# Thiazole-carboxamide derivatives as potent antioxidant agents with drug-like properties: In vitro, molecular docking, and DFT studies

**DOI:** 10.1371/journal.pone.0331000

**Published:** 2025-09-19

**Authors:** Mohammed Hawash, Dina Ghannam, Leen Dawoud, Mais Dawoud, Ahmed Eid, Lara Alhajj, Mohammed T. Qaoud

**Affiliations:** 1 Department of Pharmaceutical Chemistry and Technology, Faculty of Pharmacy, An-Najah National University, Nablus, Palestine; 2 Department of Pharmacy, Faculty of Pharmacy, Cyprus International University, Northern Cyprus, Nicosia, Türkiye; Kwara State University, NIGERIA

## Abstract

The search for novel therapeutic agents with potent antioxidant and antidiabetic properties remains a critical area of research in medicinal chemistry. Oxidative stress, caused by an imbalance between free radicals and the body’s antioxidant defenses, is implicated in numerous diseases, including diabetes, cancer, and neurodegenerative disorders. The *in vitro* evaluation of the antioxidant activity and α-amylase inhibitory potential was conducted on a series of thiazole-carboxamide derivatives, among this series, the strongest antioxidant activity against the DPPH free radical was exhibited by LMH6, with an IC₅₀ value of 0.185 µM, followed by LMH7 with an IC₅₀ value of 0.221 µM. Notably, the positive control Trolox exhibited a comparatively higher IC₅₀ value of 3.10 µM, underscoring the exceptional antioxidant potential of the synthesized compounds. Upon evaluating the inhibitory potency of the LMH series against the α-amylase enzyme, as a measure of their potential antidiabetic activity, the compounds generally exhibited modest to weak activity. In this case, their inhibition profiles were notably less potent compared to the respective positive control (acarbose). Subsequently, molecular docking studies were conducted to explore potential mechanisms that may underlie the observed antioxidant and antidiabetic activities. While these in silico analyses suggest possible interactions, particularly with the Keap1 protein, they serve as complementary hypotheses rather than direct validation of the *in vitro* findings. Docking scores, MM-GBSA binding energies, and association patterns were recorded and studied. Also, a DFT study was conducted to gain deeper insights into the free radical scavenging potential of the most potent antioxidant in the LMH series. The evaluated thiazole-carboxamide derivative demonstrated enhanced antioxidant potential by surpassing the reference compounds in terms of E_HOMO-LUMO_ gap, electron affinity (EA), and ionization potential (IP). It was also evaluated the druggability of the tested compounds using Lipinski’s Rule of Five (LRO5). This analysis helps determine their drug-like properties based on established physicochemical criteria. The analysis confirmed that all the derivatives (LMH1–LMH9) met the LRO5 criteria, indicating their potential as orally active drug candidates. The ideal drug-likeness characteristics of these derivatives support the findings, highlighting the need for further preclinical and biological studies. These molecules could greatly facilitate future therapeutic research and approval due to their beneficial properties.

## Introduction

Oxidative stress occurs in the human body when its antioxidant defenses are overwhelmed, leading to the accumulation of reactive oxygen species (ROS), including free radicals, at unhealthy levels. The ROS are highly reactive molecules that can damage cells, proteins, and DNA. They are produced naturally as a byproducts of normal cellular metabolism, but their production can also be increased by factors such as exposure to environmental toxins, radiation, and certain medications. Many diseases and ailments can develop or worsen when the equilibrium among antioxidants and oxidants is upset, a situation known as oxidative stress [1]. Managing stress, eating well, and getting plenty of exercise are all part of a healthy lifestyle that can help lower oxidative stress. Some research suggests that taking antioxidants like vitamins C and E lowers oxidative stress [2,3]. Maintaining a proper balance between oxidants and antioxidants is essential to prevent oxidative stress and reduce the risk of disease. Maintaining an active routine with consistent physical activity, a well-rounded diet, and avoiding tobacco and excessive alcohol use will assist in helping the body’s antioxidant defense systems work better and lessen oxidative stress [4,5].

Recent studies have documented the dual free radical scavenging and antidiabetic activities of various agents, particularly their inhibition of α-amylase, indicating the presence of interconnected mechanisms underlying these pharmacological effects [6–8]. α-amylase is an enzyme that hydrolyzes 1,4-linked polysaccharides, breaking down starch and other complex carbohydrates into simpler sugars, such as glucose, thus facilitating their absorption. This enzyme presents in human and some animal saliva and is also produced by certain microorganisms [9]. In light of its crucial role in regulating blood glucose levels, α-amylase inhibition has emerged as a promising strategy for managing diabetes mellitus (DM), particularly type II DM, which is marked by impaired glucose metabolism and insulin resistance [10,11].

**Fig 1:**
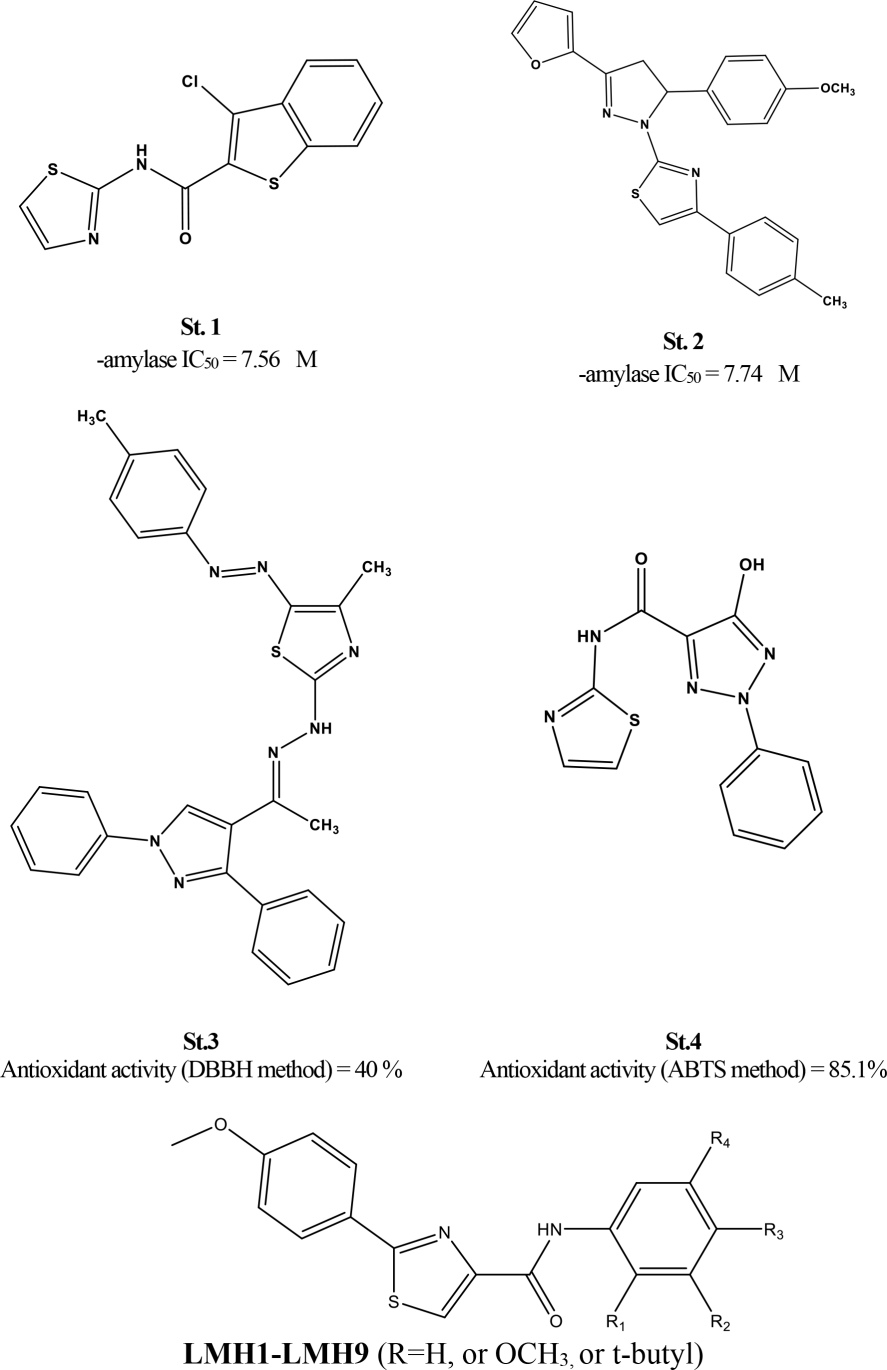
Representative structures of thiazole- and thiazole carboxamide-based agents demonstrating significant dual antioxidant and antidiabetic activities.

Heterocycles, chemical compounds with a ring structure containing at least one non-carbon heteroatom, have gained attention for their diverse pharmacological properties. Thiazole is a five-membered aromatic heterocycle containing both a nitrogen and a sulfur atom [12,13]. This promising heterocyclic scaffold has served as the core structure for various compounds exhibiting excellent and diverse pharmacological potential. It has demonstrated efficacy against a wide range of clinical conditions, including cancer, inflammation, fungal and bacterial infections, and carbonic anhydrase inhibition [14,15]. Additionally, thiazole- and thiazole carboxamide-based agents have demonstrated significant α-amylase inhibitory activity (e.g., **St.1–2**) and potential antioxidant properties (e.g., **St.3–4**), as illustrated in **Fig 1** [16–19].

Building on these promising findings, our hypothesis is to re-synthesize the previously reported thiazole-carboxamide series, which have demonstrated potent COX inhibition alongside moderate anticancer activity [20]. This provides a strong foundation for further evaluation of their antioxidant potential and inhibitory effects on α-amylase enzymes. These findings are further supported by the prevalence of agents exhibiting dual COX-inhibitory and antioxidant activities in the literature [21–23]. Additionally, comprehensive chemoinformatic studies will be conducted, including molecular docking to analyze binding profiles and optimal fitting poses within their respective target sites. HOMO-LUMO orbital energy visualization will be performed to assess electron density distribution, identify electron-rich and electron-deficient regions, and determine the HOMO-LUMO energy gap, providing insights into their free radical scavenging potential. Finally, drug-likeness assessments will be carried out to evaluate druggability profiles and potential for progression into preclinical and clinical studies.

## Materials and methods

### Chemicals

DMSO, sodium phosphate buffer (Na₂HPO₄/NaH₂PO₄, 0.02 M), NaCl (0.006 M), porcine pancreatic α-amylase enzyme solution (2 units/mL), starch solution (1% w/v in water), sodium potassium tartrate tetrahydrate, 2 M NaOH, 3,5-dinitrosalicylic acid (DNSA) solution, and distilled water (dd H₂O). Additionally, Na₂HPO₄/NaH₂PO₄, Trolox, methanol, starch, porcine pancreatic amylase, DNSA, sodium carbonate (Na₂CO₃), NaCl, dimethyl sulfoxide (DMSO, anhydrous), and acarbose were used.

### Antioxidant activity method

The free radical scavenging potential (antioxidant activity) of thiazole-carboxamide derivatives was assessed utilizing the DPPH assay. For each structure, including the positive control Trolox, A 1000 μg/mL stock solution was prepared in methanol. Subsequently, using the stock solutions, the following six serial dilutions with concentrations of 0.05, 1, 5, 50, and 100 μg/mL were prepared. After that, 0.002 g/mL of DPPH dissolved in methanol was added to each of those previously prepared dilutions. Then, a final volume of 3 ml was obtained by adding 1 ml of methanol. Due to its sensitivity to light, the DPPH solution was freshly prepared. The blank control solution for the concentration series consisted of DPPH in methanol at a 1:2 ratio, without any compound.

These steps were followed by incubating these prepared solutions at room temperature (25°C) for 30 minutes in the dark. At last, using spectrophotometer at a wavelength of 517 nm, the optical absorbance was quantified. The % of DPPH inhibition for each tested structure was measures utilizing the following equation, considering Trolox compound as the standard agent:


$$\text{DPPH}\;\text{inhibition}\;\%\;=\;(\;{\text{A}}_{\text{B}}-\;{\text{A}}_{\text{ts}})/{\text{A}}_{\text{B}}\times \;\text{100}\%$$

where, A B and Ats are is the observed absorbance value with respect to the blank and tested sample solution, respectively, this procedure was performed in triplicate [24]. The antioxidant halfmaximal inhibitory concentration (IC_50_) of the synthesized carbazole derivatives and Trolox were assessed by using an online tool “Quest Graph™ IC_50_ Calculator.”AAT Bioquest. The IC_50_ values were converted to the µM unite according to each molecule molecular weight, the raw data for DPPH assay with replications and used different concentrations for all evaluated compounds were presented in S1 Table.

### α‑Amylase inhibitory assay

The protocol started by preparing stock solutions of 1000 μg/mL concentration using 5 mg of each fraction and dissolving it in negligible volume of 10% DMSO. Then a mixture of 0.006 M NaCl and 0.02 M Na₂HPO₄/NaH₂PO₄ (pH 6.9) was used to dilute the left amount weight. After that, various concentration of 5, 50, and 100 μg/mL were prepared using both the stock and 10% DMSO solution.

To evaluate α-amylase enzyme inhibition potency, 0.2 mL of each compound dilutions was mixed with a same volume of enzyme solution (2 units/mL) and left to incubate 30°C for 10 minutes. After the end on incubation, using 0.2 ml of starch solution (1% concentration in water, freshly prepared), the mixture was rinsed and left for further 3 minutes at least. After that, the colored reagent 3,5-dinitrosalicylic acid (DNSA), 0.2 ml volume, was added to pause the reaction which followed by diluting the mixture with distilled water (5 ml). Then, in a water path, the prepared mixture was thermally treated in a water path (for 10 minutes, 90°C) which followed by allowing the solution to be cooled at R.T. After that, At the wave length 540 nm, the absorbance was quantified, this procedure was performed in triplicate,the raw data for α-amylase assay with used different concentrations for all evaluated compounds were presented in S2 Table.

The blank control was prepared by substituting 0.2 mL of buffer for the agent mixture, while the same procedure was followed using acarbose as the standard reference.

The following equation was used to determine the inhibitory activity of α-amylase:


$$\%\;\text{of}\;\alpha -\text{amylase}\;\text{inhibition}\;=\;({\text{A}}_{\text{B}}\;-\;{\text{A}}_{\text{T}})/{\text{A}}_{\text{B}}\;\times \;\text{100}\%,$$

where, ${\text{A}}_{\text{B}}$ and ${\text{A}}_{\text{T}}\;$are the absorbance of the blank and the test sample, respectively [25].

### Chemoinformatic studies

#### Molecular docking study.

A molecular docking study was undertaken to explore the binding modes of thiazole-carboxamide derivatives when docked to protein’s binding domain, thereby elucidating the mechanism of action underlying their observed *in vitro* assay. The research methodology followed a systematic protocol encompassing ligand preparation and sketching, receptor preparation, identifying the receptor’s grid box and final XP-Glide docking simulations that were performed in accordance with these established protocols. These steps were carried out using the Maestro Graphical User Interface and the integrated modules of the Maestro Schrödinger Suite (version 14.2), including LigPrep, Protein Preparation Wizard, Receptor Grid Generation, and Glide docking, respectively. This integrated approach ensured consistency and reproducibility throughout the computational workflow.

***Ligand Preparation and Sketching*:** The initial stage involved employing the Maestro graphical user interface to construct and visually model the selected ligands in accordance with the OPLS2005 force field parameters through a comprehensive preparation process using the LigPrep module. To ensure a realistic representation of their biological conditions, the ligands were carefully adjusted to their corresponding protonation states at a physiological pH of 7.0 ± 2.0 [26].

***Protein preparation and grid generation***. The Kelch (Keap1) co-crystal structure (PDB ID: 2FLU, Resolution: 1.50 Å) was utilized to investigate the antioxidant activity of the synthesized derivatives. The human pancreatic α-amylase (PDB ID: 4W93, Resolution: 1.35 Å) co-crystal structure, presents as complex with montbretin A ligand, was used to assess α-amylase inhibition. These crystallographic structures were retrieved from the free server Protein Data Bank (https://www.rcsb.org/) and systematically optimized using Protein Preparation Wizard. This step embedded several critical sub steps such as adding hydrogen atoms and adjusting charge states and bond orders aiming to simulate the chemical and biological conditions, making it ideal for optimizing our synthesized derivatives. The effectiveness of this approach is further validated by the strong correlation between docking scores and bioactivity, as demonstrated by previous studies [27]. To facilitate accurate molecular docking simulations, receptor grids were generated with precise dimensions of 20 Å × 20 Å × 20 Å for all utilized crystallographic structures. As the crystallographic structure of α-amylase (PDB ID: 4W93) was retrieved as complexes with their native ligands, the receptor grid was centered on these native ligand to ensure optimal coverage of the active binding site. For Keap1, the active site responsible for protein activation—including key residues such as Ile416, Ala607, Val608, Gly367, and Val418—was identified based on literature evidence [28].

***Glide extra-precision (XP) ligand docking:*** A highly precise XP-Glide docking procedure was performed. The Van der Waals scaling factors and partial charge cutoffs were meticulously set to 0.80 and 0.15 for ligand atoms, respectively, to ensure accuracy in the docking process [29]. After energy minimization, Glide scoring was used to evaluate the best binding conformations. For each docked ligand, the conformation with the best Glide docking score was selected as the optimal binding pose. These binding modes were further analyzed in detail using the PLIP server [30].

### Calculations of free energy using prime MM-GBSA

The Prime Molecular Mechanics—Generalized Born Surface Area (Prime MM-GBSA) module within the Maestro Schrödinger interface (version 14.2) was employed to predict the binding energies of the docked ligands within the binding sites of target proteins. The calculations utilized the VSGB 2.0 (2021) model for solvent effects, and the OPLS4 force field was applied for accurate energy estimation. The total free energies of all ligand-receptor complexes were systematically recorded. Thermodynamic equations were then applied to determine the variations in free energy:


$${\text{G}}_{\text{complex}}\;-\;({\text{G}}_{\text{protein}}\;+\;{\text{G}}_{\text{ligand}})\;=\;-\Delta {\text{G}}_{\text{binding}}$$ Eq. 1

The ligand binding energy is denoted as ΔGbind in this equation, and the optimized energies of the free protein, the free ligand, and the protein-ligand complex are denoted by Gprotein, Gligand, and Gcomplex, respectively [31].

### DFT analysis

The Maestro Schrödinger interface (version 14.2) was used to perform the DFT analysis utilizing a module named **Jaguar-Single Point Energy** module. The calculations employed the B3LYP-D3 functional with the 6-31G** basis set. Input files were prepared within Jaguar, and the output was formatted in Gaussian-compatible format for further analysis. The medium grid density, fast accuracy level, and maximum iterations were applied as parameters for the calculations [32,33]. Key descriptors, such as the atomic electrostatic potential (ESP) map surface, ionization potential (IP), electron affinity (EA), HOMO-LUMO gap (ΔE), the lowest unoccupied molecular orbital (E_LUMO_), and the highest occupied molecular orbital (E_HOMO_), were simultaneously evaluated [34,35].

### Statistical analysis

To compare multiple means, an analysis of variance (ANOVA) was conducted using GraphPad Prism. Data are presented as the mean ± standard deviation. The antioxidant, and antidiabetic procedures for the evaluated compounds were performed in triplicate, pairwise comparisons between the groups and the control were performed using ANOVA followed by t-tests. A *p*-value of less than 0.05 was considered statistically significant for the evaluation.

## Results and discussion

### Chemistry

The chemical structures of the synthesized thiazole-carboxamide derivatives (LMH series) are summarized in Fig 1 and Table 1. To activate and couple reactions, both EDCI and DMAP were utilized. The HRMS and 1H-NMR validating tools were utilized to check the synthetic derivatives’ chemical compositions [36].

**Table 1:** IC_50_ values (µM) of Thiazole-Carboxamide Compounds and Positive Controls on DPPH, and Percentage of Inhibition of α-Amylase.

Code	R1	R2	R3	R4	DPPH IC_50_ (µM) ±SD	% of inhibition of α-Amylase ±SD at 100 µg/mL
LMH1	H	-O-CH_3_	-O-CH_3_	H	0.316 ± 0.040	41.81 ± 2.01
LMH2	H	-O-CH_3_	-O-CH_3_	-O-CH_3_	2.462 ± 0.98	47.95 ± 1.88
LMH3	H	H	H	H	1.085 ± 0.402	27.13 ± 2.53
LMH4	H	-O-CH_3_	H	-O-CH_3_	0.251 ± 0.057	37.71 ± 0.28
LMH5	-O-CH_3_	H	Cl	-O-CH_3_	1.662 ± 0.192	36.34 ± 1.89
LMH6	H	H	t-butyl	H	0.185 ± 0.049	22.69 ± 3.11
LMH7	-O-CH_3_	H	H	-O-CH_3_	0.221 ± 0.059	24.74 ± 2.73
LMH9	-O-CH_3_	H	-O-CH_3_	H	0.618 ± 0.081	28.83 ± 2.43
+ve control	–	–	–	–	3.10 ± 0.92^a^	77.87 ± 0.85^b^

Note: ^a^Trolox, ^b^Acarbose; *P* value ≤ 0.05

### Biological evaluations

#### Antioxidant activity.

One popular way to measure a chemical’s antioxidant activity *in vitro* is utilizing the 2,2-Diphenyl-1-picrylhydrazyl (DPPH) agent. This assay measures the capability of the tested agents to scavenge DPPH radicals, which are stable and synthetic radicals that have a violet color. When DPPH reacts with an antioxidant compound, the radical is neutralized, alongside detecting a change in the color of the DPPH from violet to yellow. This color change can be measured using a spectrophotometer, and the extent of this change is proportional to antioxidant strength of the compound being tested. The DPPH assay is a simple, rapid, as well as sensitive method for assessing the antioxidant activity of a wide range of compounds, including vitamins, polyphenols, and other natural and synthetic compounds [37]. Herein, the evaluation of the DPPH reduction ability, serving as an indicator of antioxidant activity, was conducted for the synthesized thiazole-carboxamide derivatives. The antioxidant activity of the synthesized thiazole-carboxamide derivatives was evaluated by their ability to scavenge DPPH free radicals, as determined by the decrease in absorbance at 517 nm. As summarized in Table 1, the IC₅₀ values were calculated and expressed as mean ± standard deviation (SD) from triplicate measurements.

The evaluated compounds displayed strong to moderate free radical scavenging potential, with several outperforming the standard antioxidant, Trolox (IC₅₀ = 3.10 ± 0.92 µM). Notably, LMH6 and LMH7 exhibited the most potent activity, with IC₅₀ values of 0.185 ± 0.049 µM and 0.221 ± 0.059 µM, respectively. These values were statistically significantly lower than that of Trolox (P ≤ 0.05), indicating markedly enhanced antioxidant properties. Additionally, compounds LMH4 (0.251 ± 0.057 µM) and LMH1 (0.316 ± 0.040 µM) also demonstrated potent scavenging activity, suggesting a possible structure–activity relationship influenced by methoxy and bulky alkyl substituents. The narrow SD values across all tested compounds confirm the reliability and reproducibility of the assay data.

The DPPH assay is a useful method for evaluating antioxidant potential but has limitations, as it only measures free radical scavenging through hydrogen or electron donation. It does not reflect other mechanisms like enzymatic activity or cellular ROS scavenging. Therefore, further studies using assays such as ABTS, FRAP, or cell-based models are recommended for a more comprehensive assessment of antioxidant efficacy and biological relevance [38].

While antioxidant activity provides valuable insight into the potential of compounds to counter oxidative stress, other biological targets relevant to disease management also warrant investigation. One such target is α-amylase, an enzyme closely linked to postprandial hyperglycemia in diabetes. Therefore, the α-amylase inhibitory potential of the synthesized compounds was subsequently examined to assess their antidiabetic prospects.

#### Anti-α-amylase activity.

**Fig 2:**
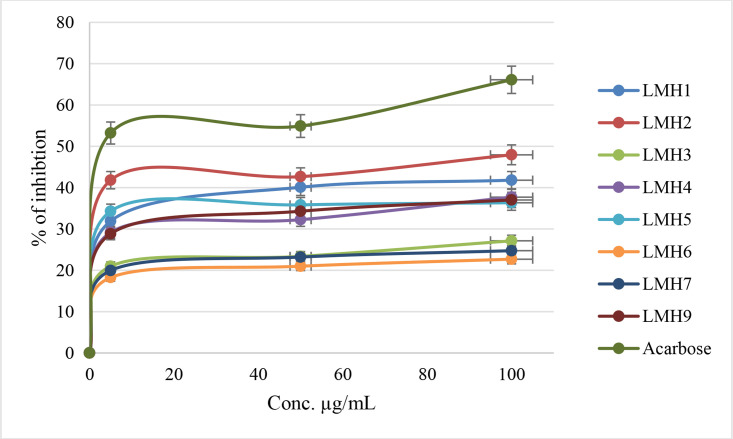
% of inhibition of thiazole-carboxamide derivatives against α-Amylase.

All compounds showed moderate to weak α-amylase inhibitory activity compared to the positive control, Acarbose (77.87 ± 0.85% inhibition at 100 µg/mL). As shown in Fig 2 and Table 1, the percentage inhibition among the LMH series ranged from 22.69 ± 3.11% for LMH6 to 47.95 ± 1.88% for LMH2 at a concentration of 100 µg/mL. Although LMH2 exhibited the highest inhibition among the tested compounds, it remained significantly lower than that of the standard (P ≤ 0.05).

The standard errors associated with the inhibition values indicate acceptable repeatability, and the relatively narrow margins confirm the reliability of the trends observed. While no compound approached the potency of Acarbose, several (e.g., LMH1, LMH2, LMH4) showed moderate activity, suggesting potential for further optimization. These findings are consistent with the structural variations, particularly the number and position of methoxy substituents, which appear to influence enzyme binding affinity.

All compounds showed moderate to weak α-amylase inhibitory activity compared to the positive control (Acarbose). Referred to the results shown in Fig 2, the percentage of α-amylase inhibition among the LMH series ranged from 22.69% for LMH6 to 47.95% for LMH2 at a concentration of 100 µg/mL.

To gain deeper insights into the molecular basis of the observed antioxidant and enzyme inhibition activities, molecular docking studies were undertaken. These simulations allow for the exploration of binding modes, key interactions, and the structural features responsible for biological activity, helping to rationalize the experimental findings and guide future optimization.

### Chemoinformatic analysis

#### Molecular docking study.

**Fig 3:**
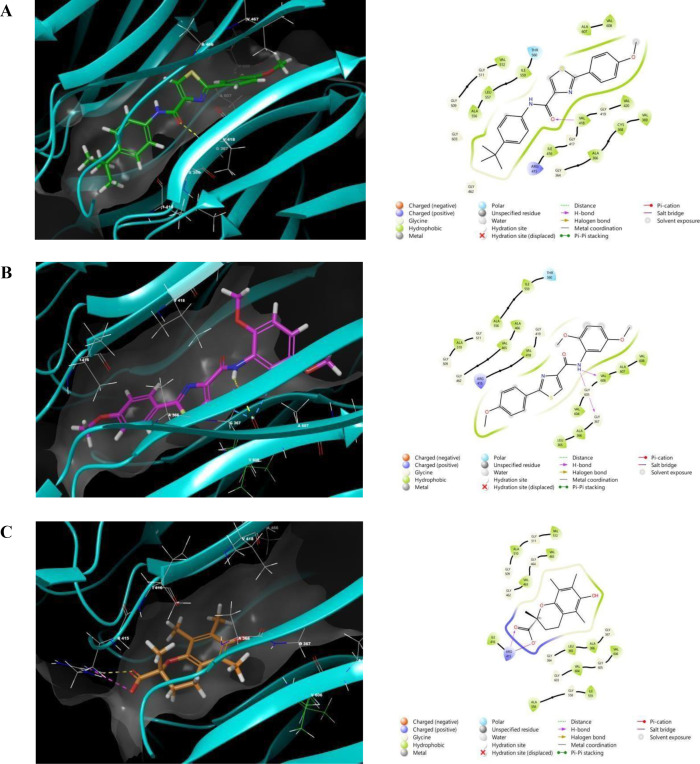
Molecular docking simulations of LMH6 (A), LMH7 (B), and Trolox (C) within the binding site of the Keap1 protein (PDB ID: 2FLU). Yellow, magenta, and blue represent hydrogen bonds, salt bridges, and aromatic hydrogen bonds, respectively.

**Fig 4:**
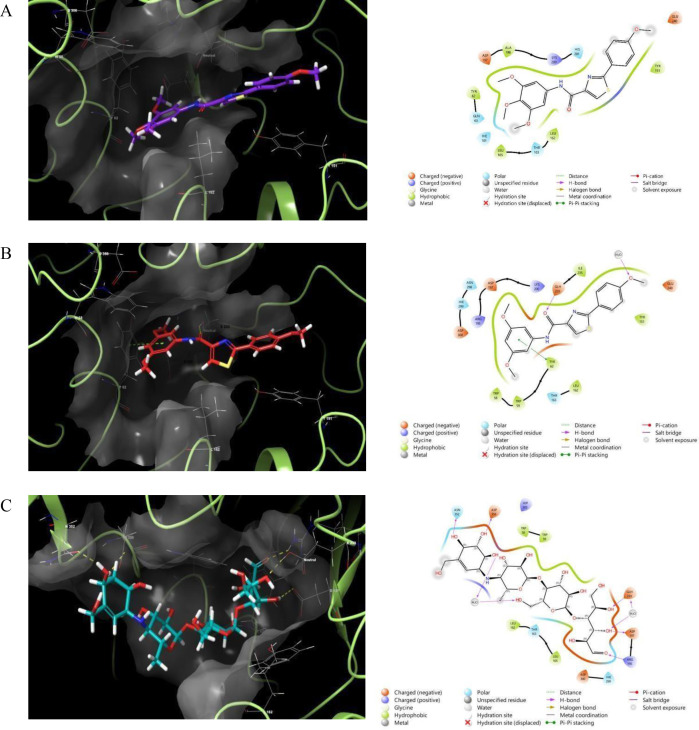
Molecular docking simulations of LMH2 (A), LMH4 (B), and acarbose (C) within the binding site of the Human pancreatic alpha-amylase (PDB ID: 4W93). Yellow, magenta, and blue represent hydrogen bonds, salt bridges, and aromatic hydrogen bonds, respectively.

The most active thiazole-carboxamide compounds were studied using molecular docking models to find their binding contacts and optimal matching configurations with important therapeutic targets, such as Keap1 and α-amylase, as shown in **Figs 3 and 4**, correspondingly. For the purpose of designing and optimizing new bioactive compounds, docking experiments are an effective analytical method in drug development because they provide important insights into atomic-level molecular recognition and interactions [39]. The detailed descriptions of each compound’s encounters, comprising the creation of hydrogen bonds, hydrophobic interactions, and π-cationic interactions, are outlined in **Table 2**. Additionally, docking scores and MM-GBSA binding energies for each tested agent are provided for evaluating the strength of the binding affinity. Binding interactions can be clarified with the help of these

**Table 2:** The Interaction profiles, docking scores, and binding energies of the tested chemical structures within their respective binding domains.

Target	Name	H. Bs	HPHO	π- Cationic	Docking Score (kcal/mol)	MM-GBSA (ΔG)
**Keap1** **(PDB ID: 2FLU)**	**LMH6**	**VAL418**	**ALA366, VAL420,** **ALA607**	**–**	**−7.11**	**−63.80**
	**LMH7**	**VAL606, GLY367**	**ALA607**	**–**	**−6.87**	**−67.00**
	**Trolox**	**ARG415**	**–**	**–**	**−6.22**	**−39.17**
**α-Amylase** **(PDB ID: 4W93)**	**LMH2**	**–**	**TYR151, LEU162**	**–**	**−4.27**	**−37.93**
	**LMH4**	**GLH233**	**TRP58, ILE235**	**TYR62**	**−5.33**	**−44.86**
	**Acarbose**	**GLH233, ASP197, ARG195, ASP356,** **ASN352,**	**–**	**–**	**−6.15**	**−50.22**

quantitative investigations, which can direct the development and testing of these compounds in the lab for potential medicinal uses [25].

Initially, to ensure the reliability of our docking methodology, multiple validation strategies were employed. For Keap1, the binding interactions of the reference ligand Trolox were compared with literature data and exhibited an identical interaction profile, confirming the reproducibility of our protocol [28]. In the case of α-amylase, the docking approach was rigorously validated in our prior published studies, and the current work extends these established methods [25,40,41]. Collectively, these validation approaches—literature alignment and precedent methodological consistency—affirm the accuracy and robustness of our docking protocols and computational workflow.

The potential of **LMH6** and **LMH7**, which exhibited the strongest antioxidant profiles, alongside the positive control Trolox was evaluated for their ability to interact with the Keap1 protein binding domain to assess their role in facilitating Nrf2 dissociation. Nuclear factor erythroid 2-related factor 2 (Nrf2) is a crucial transcription factor that regulates cellular defense mechanisms against oxidative stress. Previous studies have established a strong correlation between ligand-induced conformational changes in Keap1 and subsequent Nrf2 activation. This activation promotes the translocation of Nrf2 into the nucleus, where it binds to the antioxidant response element (ARE), thereby enhancing the expression of cytoprotective genes and fortifying cellular resilience against oxidative damage [42,43]. As observed, both **LMH6** and **LMH7** exhibited notable binding affinities, with docking scores of −7.11 and −6.87 kcal/mol, respectively. **LMH6** formed key hydrogen bonds with VAL418 while engaging in hydrophobic interactions with surrounding residues such as ALA366, VAL420, and ALA607. In contrast, **LMH7** formed two favorable hydrogen bonds with VAL606 and GLY367, alongside a hydrophobic interaction with ALA607, highlighting distinct but significant binding characteristics. Trolox, the positive control, displayed a slightly weaker affinity with a docking score of −6.22 kcal/mol and a binding energy of −39.17 kcal/mol. Returning to **Fig 3**, both **LMH6** and **LMH7** showed better fitting profiles with respect to their occupied space and interactions within the binding site. These results suggest that **LMH6** and **LMH7** have substantial potential for Keap1 binding, with **LMH6** demonstrating a stronger overall affinity based on its docking score and binding energy.

The docking simulations for α-amylase (PDB ID: 4W93) revealed that **LMH4** exhibited the highest affinity among the tested compounds, with a docking score of −5.33 kcal/mol and an MM-GBSA binding energy of −44.86 kcal/mol. This compound formed significant interactions, including hydrogen bonds with GLH233, π-π stacking interactions with TYR62, and hydrophobic interactions with surrounding residues such as TRP58 and ILE235. **LMH2**, with a docking score of −4.27 kcal/mol, formed hydrophobic interactions with TYR151 and LEU162. Acarbose, the positive control, exhibited the highest docking affinity with a score of −6.15 kcal/mol and a binding energy of −50.22 kcal/mol, which may explain the lower inhibition potency of **LMH2** and **LMH4** compared to acarbose. This is further supported by the docking poses depicted in **Fig 4** and the favorable fitting profiles of **LMH4** and **LMH2** within the target’s binding domain, suggesting moderate efficacy as α-amylase inhibitors.

The favorable interaction profiles observed across the tested targets—Keap1–Nrf2 and α-amylase—highlight promising pharmacological implications. Targeting the Keap1–Nrf2 pathway is a well-validated strategy for enhancing cellular antioxidant defenses [44]. Compounds such as LMH6 and LMH7, which mimic the action of the reference antioxidant Trolox, demonstrated the ability to disrupt the Keap1–Nrf2 complex. This disruption can upregulate cytoprotective genes, offering therapeutic potential in the management of oxidative stress-related disorders such as neurodegenerative diseases, diabetes, and cancer [45]. The strong binding affinities of LMH6 and LMH7 suggest their potential as lead compounds for the development of novel Nrf2 pathway activators. Likewise, α-amylase plays a critical role in carbohydrate metabolism by catalyzing the hydrolysis of starch to glucose, making it a key target in controlling postprandial blood glucose levels in type 2 diabetes mellitus [46]. Although the LMH series exhibited only mild to weak inhibitory potency and less optimal interactions compared to acarbose, the observed binding patterns support their potential as lead structures for the development of α-amylase inhibitors with improved therapeutic profiles and reduced gastrointestinal side effects.

In summary, the molecular docking study indicates that the tested thiazole-carboxamide derivatives exhibit promising binding affinities and interactions across different targets. **LMH6** and **LMH7** demonstrate significant potential as Keap1 activators, while **LMH4** and **LMH2** show substantial inhibitory activity against α-amylase, respectively. These findings suggest that these compounds may serve as promising candidates for further development as therapeutic agents for a range of diseases, including cancer and diabetes, based on their robust binding profiles and favorable drug-like properties.

To further support the *in vitro* and docking results, DFT analysis was performed to explore key electronic properties to better understand orbital behavior, charge transfer, and reactivity. These insights help explain the compounds’ bioactivity and complement the molecular interaction findings.

#### DFT analysis.

**Fig 5:**
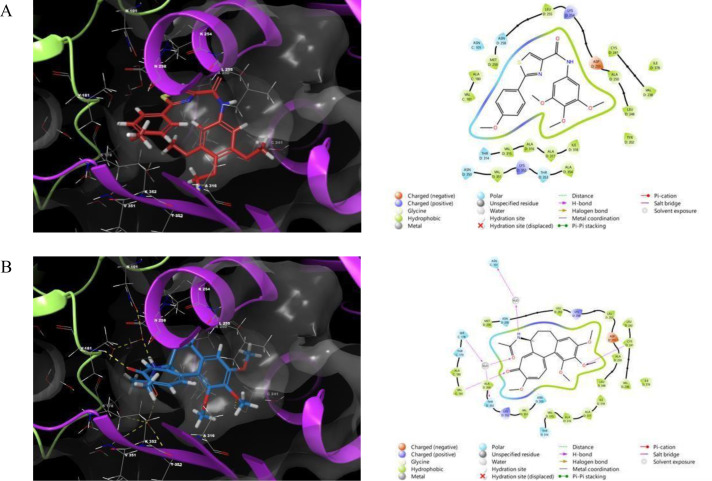
The 3D highest occupied molecular orbital (HOMO) and lowest unoccupied molecular orbital (LUMO) profiles of LMH6 and Trolox structures.

The distribution patterns of the frontier molecular orbitals (FMOs), namely the highest occupied molecular orbital (HOMO) and the lowest unoccupied molecular orbital (LUMO), for all tested compounds are depicted in **Fig 5**. Orbitals in question have a high energy level, which indicates that they can donate or take electrons, particularly when they are antibonding. In particular, electrophilic attacks frequently target the HOMO because of its electron-rich surroundings, which is ideal for such interactions. However, the LUMO is a prime target for nucleophilic assault because of its electron-deficient nature and its capacity to receive electron density. Within each of the molecules that were examined, there was a demonstration of intramolecular charge transfer (ICT) from the HOMO to the LUMO.

Analysis of the HOMO-LUMO maps reveals distinct features in the distribution of the orbitals. For **LMH6** compound, the HOMO orbitals are primarily located over the phenyl-amide motifs, whereas for Trolox, the HOMO is concentrated over the carboxylic acid functional group. On the other hand, the LUMO orbitals for **LMH6** are predominantly localized over the thiazole ring, while in Trolox, the LUMO orbitals are allocated over the chromane ring. This suggests that the electronic characteristics of these compounds are influenced by their structural motifs, which in turn affects their potential for interaction with target sites.

In **Table 3**, the statistics calculated at the B3LYP/6-31G** level of theory highlight the HOMO and LUMO energy levels, energy gaps (Egap), ionization potential (IP), and electron affinity (EA). Importantly, the investigated compound (**LMH6**), that demonstrated the most potent antioxidant activity, has electron donation ability that is similar to Trolox, as its E_HOMO_ energy level is comparable. This chemical structure may be better able to receive electrons since its E_LUMO_ level energy is significantly lower than Trolox’s. The molecule under study has an electrical configuration that is advantageous for interaction with locations of interest, as its Egap value is less than Trolox’s.

**Table 3:** Descriptors obtained from DFT analysis.

	E_HOMO_	E_LUMO_	E_gap_	IP	EA
**LMH-6**	**−5.64**	**−1.60**	**4.04**	**8.56**	**1.28**
**Trolox**	**−5.42**	**0.12**	**5.54**	**8.9**	**0.12**

Concerning charge transfer properties, the higher EA value of **LMH6** compound compared to Trolox suggests an enhanced electron transport ability. The scavenging potential for free radicals can be assessed via single electron donation, where IP serves as an important descriptor to evaluate the electron transfer range. By removing an electron from the HOMO, a one-electron transfer radical cation can be generated. **Table 3** shows that the studied compound has smaller IP value compared to Trolox, implying that this compound may exhibit more efficient electron transfer mechanisms, which could result in superior free radical scavenging activity compared to the reference compound, Trolox.

Aiming to understand the molecular reactivity and potential interaction sites more, molecular electrostatic potential (MEP) surfaces were subsequently visualized. MEP analysis complements DFT by revealing regions prone to nucleophilic or electrophilic attack, which aids in predicting biological behavior and target interactions.

#### Electrostatic potential.

**Fig 6:**
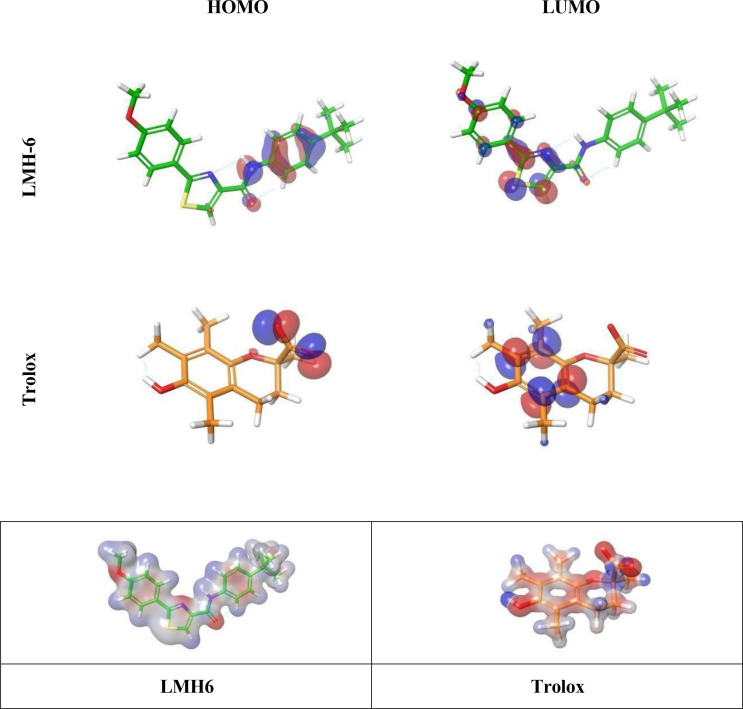
Electrostatic potential profiles of LMH6 and Trolox molecules, depicted as solid surfaces.

An invaluable resource for comprehending molecular interactions and locating comparative sensitivity regions for electrophilic and nucleophilic assaults are the three-dimensional (3D) surface maps that represent the molecular electrostatic potential (MEP). In **Fig 6**, the MEP surface maps of **LMH6** and Trolox are illustrated to highlight the distribution of electrostatic potential (ESP) regions, providing insights into their electronic environments.

The MEP panels use a colour-coded system to show various ESP areas; zones with a neutral potential are white, regions with a potential that is positive are blue, and areas with a negative potential are red. The ones with a negative electrostatic potential (red) are more likely to be affected by electrophiles, while those with a positive ESP (blue) are more likely to be affected by nucleophiles [33]. These differences in electrostatic potential provide crucial information about possible reaction areas.

In our analysis, the positive electrostatic potential is predominantly localized around terminal carbon groups, whereas the negative potential is concentrated on oxygen- and nitrogen-containing functional groups. This distribution suggests that key reactive sites in **LMH6** are likely influenced by their oxygen and nitrogen moieties, which play a crucial role in determining its molecular interactions and potential biological activity. The comparative analysis with Trolox further highlights how structural differences contribute to variations in electrostatic potential, potentially impacting the binding affinity and reactivity of these compounds.

Beyond activity and reactivity, assessing the drug-likeness of candidate molecules is essential for evaluating their pharmacokinetic feasibility. Therefore, in silico drug-likeness prediction tools were employed to evaluate whether the synthesized compounds possess favorable properties for oral bioavailability and further development as therapeutic agents.

#### Drug-likeness analysis.

Previously, the druggability profiles of thiazole carboxamide derivatives were evaluated using computational techniques, demonstrating optimal results across a set of physicochemical parameters [36]. To further support these findings, this study conducted drug likeness model tests to assess their drug-likeness capability. As observed in Table 4 and S1 Fig.

**Table 4:** Drug-likeness model’s score for Thiazole-carboxamide compounds.

Compound	LMH1	LMH2	LMH3	LMH4	LMH5	LMH6	LMH7	LMH9
Score	−0.11	0.66	0.14	0.37	0.73	0.02	0.21	−0.04

#### SAR analysis.

The previously tested thiazole-carboxamide compounds, initially designed as COX inhibitors, herein demonstrated potent antioxidant activity, with IC₅₀ values below 2.462 ± 0.98 µM. Among them, **LMH6** exhibited the highest activity, with an IC₅₀ of 0.185 ± 0.049 µM (**Table 1**). This highlights the crucial role of the t-butyl group at the para position, which enhances antioxidant potency via multiple pathways. First, the t-butyl group, being highly lipophilic, improves the compound’s ability to penetrate biological membranes, allowing better interaction with lipid radicals in lipid peroxidation pathways. Additionally, the t-butyl group exhibits a strong inductive electron-donating effect, increasing the electron density on the phenyl ring. This effect stabilizes the radical formed after hydrogen donation, thereby enhancing the antioxidant activity.

Replacing the para t-butyl group with 2,5-dimethoxy groups (**LMH7**) resulted in a slight decrease in antioxidant activity, though the potency remained comparable to **LMH6**. However, introducing a chlorine substitution at position 4 alongside 2,4-dimethoxy groups (**LMH5**) had a negative effect on activity.

Compounds with 2,4-dimethoxyphenyl (**LMH9**), 3,4-dimethoxy (**LMH1**), or 3,4,5-trimethoxy substitutions (**LMH2**) showed a dramatic decrease in activity, indicating the negative impact of methoxy substitution at the para position. Interestingly, removing all substituents and using a plain phenyl ring (**LMH3**) resulted in enhanced activity compared to para-methoxy-substituted analogs. However, placing methoxy groups at both meta positions (**LMH4**) led to moderate antioxidant activity, though it remained lower than that of **LMH6** or **LMH7**.

## Conclusion

This work assessed the *in vitro* antioxidant and antidiabetic profiles for a set thiazole-carboxamide derivatives (**LMH1–LMH9)**. The evaluated compounds exhibited significant antioxidant activity, demonstrating a strong capacity to neutralize free radicals in the DPPH assay, while showing weaker inhibitory effect against the α-amylase enzyme. Among them, **LMH6** displayed the highest antioxidant potential, followed by **LMH7**, while **LMH2** exhibited the weakest activity. Moreover, the antioxidant and therapeutic potential of these thiazole-carboxamide series were elucidated using computational approaches, including molecular docking, FMO analysis, and MEP mapping. Docking simulations indicated that **LMH6** and **LMH7** demonstrated robust bonds to Keap1, implying its capacity to promote Nrf2 activation, a key pathway in oxidative stress defense. Furthermore, **LMH4** as well as **LMH2** exhibited α-amylase inhibition activity, though at a lower level than the positive control acarbose. Importantly, DFT-based descriptors such as the Egap, IP, and EA revealed that LMH6 possesses favorable electronic properties supporting its superior antioxidant behavior. The smaller Egap and lower IP compared to Trolox suggest more efficient electron transfer, while the higher EA indicates enhanced radical scavenging potential. Complementing these findings, the MEP surface maps highlighted reactive oxygen- and nitrogen-containing regions as key electrophilic and nucleophilic sites, offering a molecular-level explanation for the compound’s reactivity and observed biological performance.

Given the promising antioxidant activity and strong Keap1 binding affinities demonstrated, particularly by LMH6 and LMH7 compounds, further preclinical and biological investigations are necessary. These should encompass *in vivo* pharmacokinetic profiling, toxicity assessments, and efficacy evaluations in relevant disease models to comprehensively validate their safety and therapeutic potential. Such studies are essential for advancing these compounds toward clinical application.

## Supporting information

S1 Fig Drug-likeness model for Thiazole-carboxamide compounds LMH 1 (a), LMH 2 (b), LMH 3 (c), LMH 4 (d), LMH 5 (e), LMH 6 (f), LMH 7 (g), and LMH 9 (h). (DOCX)

S1 Table The raw data for DPPH assay with replications and used different concentrations for all evaluated compounds. (DOCX)

S2 Table The raw data for alpha amylase assay with used different concentrations for all evaluated compounds. (DOCX)
