# Supplementary material for: Thiazole-carboxamide derivatives as potent antioxidant agents with drug-like properties: In vitro, molecular docking, and DFT studies
Source: PLoS One. 2025 Sep 19;20(9):e0331000. doi: 10.1371/journal.pone.0331000 (PMC12448349; doi:10.1371/journal.pone.0331000)
Supplement: S1 Fig — (DOCX) [file pone.0331000.s001.docx]

**S1 Fig.** Drug-likeness model for Thiazole-carboxamide compounds LMH 1 (a), LMH 2 (b), LMH 3 (c), LMH 4 (d), LMH 5 (e), LMH 6 (f), LMH 7 (g), and LMH 9 (h).

| 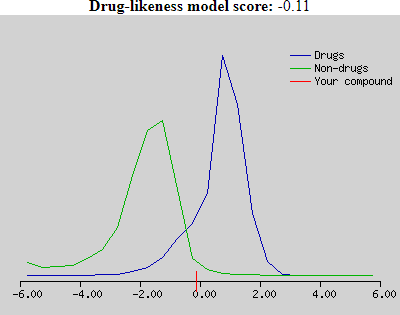 | 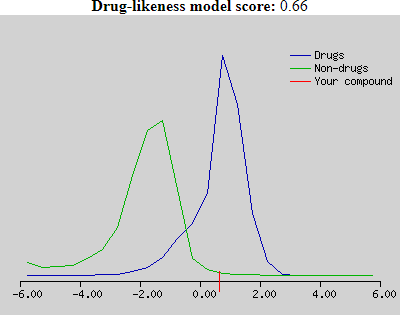 |
| --- | --- |
| **(a)** | **(b)** |
| 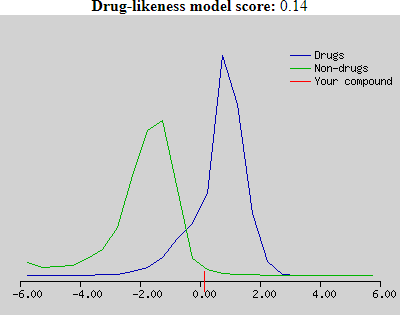 | 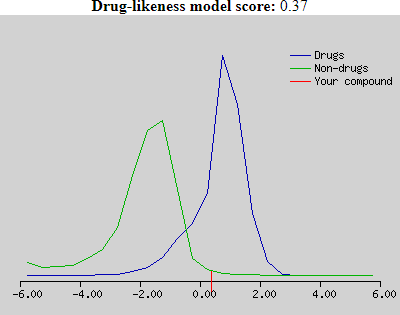 |
| **(c)** | **(d)** |
| 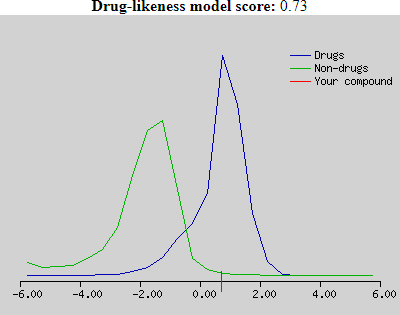 | 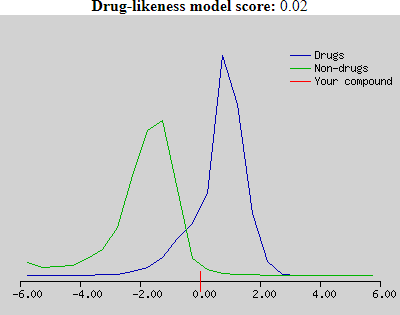 |
| **(e)** | **(f)** |
| 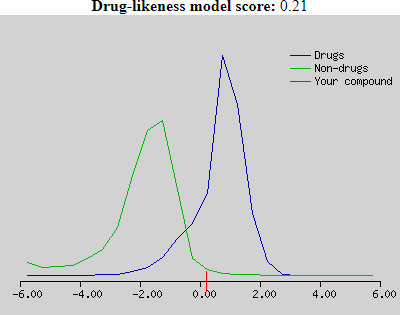 | 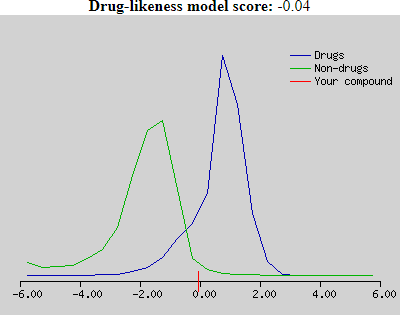 |
| **(g)** | **(h)** |
