# Supplementary material for: Thiazole-carboxamide derivatives as potent antioxidant agents with drug-like properties: In vitro, molecular docking, and DFT studies
Source: PLoS One. 2025 Sep 19;20(9):e0331000. doi: 10.1371/journal.pone.0331000 (PMC12448349; doi:10.1371/journal.pone.0331000)
Supplement: S1 Table — (DOCX) [file pone.0331000.s002.docx]

**S1 Table.** The raw data for DPPH assay with repications and used different concentrations for all evaluated compound

| LMH1 | Blank0.246 |  | MWt | 370.42 |  |  |  |  |  |  |  |  |
| --- | --- | --- | --- | --- | --- | --- | --- | --- | --- | --- | --- | --- |
| Conc. µg/mL | Abc1 | Abc2 | Ab3 | ınh%1 | ınh%2 | ınh%3 | SD inh | IC50-1 | IC50-2 | IC50-3 | Av IC50 µg/mL | SD |
| 0 | 0 | 0 | 0 | 0 | 0 | 0 | 0 | 0.103 | 0.115 | 0.133 | 0.117 | 0.0151 |
| 0.05 | 0.126 | 0.13 | 0.131 | 48.78049 | 47.15447 | 46.74797 | 1.075509 |  |  |  | IC50 µM | SD |
| 1 | 0.11 | 0.115 | 0.119 | 55.28455 | 53.25203 | 51.62602 | 1.833028 |  |  |  | 0.315857675 | 0.040764 |
| 5 | 0.105 | 0.108 | 0.115 | 57.31707 | 56.09756 | 53.25203 | 2.086017 |  |  |  |  |  |
| 50 | 0.102 | 0.104 | 0.11 | 58.53659 | 57.72358 | 55.28455 | 1.692411 |  |  |  |  |  |
| 100 | 0.095 | 0.099 | 0.105 | 61.38211 | 59.7561 | 57.31707 | 2.046026 |  |  |  |  |  |
|  |  |  |  |  |  |  |  |  |  |  |  |  |
| LMH2 | Blank0.246 |  | MWt | 400.45 |  |  |  |  |  |  |  |  |
| Conc. µg/mL | Abc1 | Abc2 | Ab3 | ınh%1 | ınh%2 | ınh%3 | SD inh | IC50-1 | IC50-2 | IC50-3 | Av IC50 µg/mL | SD |
| 0 | 0 | 0 | 0 | 0 | 0 | 0 | 0 | 1.399 | 0.941 | 0.619 | 0.986333333 | 0.391971 |
| 0.05 | 0.239 | 0.201 | 0.195 | 2.845528 | 18.29268 | 20.73171 | 9.699474 |  |  |  | IC50 µM | SD |
| 1 | 0.129 | 0.12 | 0.101 | 47.56098 | 51.21951 | 58.94309 | 5.810781 |  |  |  | 2.463062388 | 0.978827 |
| 5 | 0.12 | 0.112 | 0.095 | 51.21951 | 54.47154 | 61.38211 | 5.189896 |  |  |  |  |  |
| 50 | 0.103 | 0.09 | 0.085 | 58.13008 | 63.41463 | 65.44715 | 3.777062 |  |  |  |  |  |
| 100 | 0.099 | 0.073 | 0.071 | 59.7561 | 70.3252 | 71.13821 | 6.349796 |  |  |  |  |  |
|  |  |  |  |  |  |  |  |  |  |  |  |  |
|  |  |  |  |  |  |  |  |  |  |  |  |  |
| LMH3 | Blank0.246 |  | MWt | 310.37 |  |  |  |  |  |  |  |  |
| Conc. µg/mL | Abc1 | Abc2 | Ab3 | ınh%1 | ınh%2 | ınh%3 | SD inh | IC50-1 | IC50-2 | IC50-3 | Av IC50 µg/mL | SD |
| 0 | 0 | 0 | 0 | 0 | 0 | 0 | 0 | 0.364 | 0.446 | 0.202 | 0.337333333 | 0.124167 |
| 0.05 | 0.14 | 0.138 | 0.135 | 43.08943 | 43.90244 | 45.12195 | 1.023013 |  |  |  | IC50 µM | SD |
| 1 | 0.116 | 0.118 | 0.115 | 52.84553 | 52.03252 | 53.25203 | 0.620945 |  |  |  | 1.086874805 | 0.40006 |
| 5 | 0.097 | 0.101 | 0.097 | 60.56911 | 58.94309 | 60.56911 | 0.938781 |  |  |  |  |  |
| 50 | 0.088 | 0.095 | 0.091 | 64.22764 | 61.38211 | 63.00813 | 1.427595 |  |  |  |  |  |
| 100 | 0.074 | 0.081 | 0.069 | 69.9187 | 67.07317 | 71.95122 | 2.45029 |  |  |  |  |  |
|  |  |  |  |  |  |  |  |  |  |  |  |  |
|  |  |  |  |  |  |  |  |  |  |  |  |  |
| LMH4 | Blank0.246 |  | MWt | 370.42 |  |  |  |  |  |  |  |  |
| Conc. µg/mL | Abc1 | Abc2 | Ab3 | ınh%1 | ınh%2 | ınh%3 | SD inh | IC50-1 | IC50-2 | IC50-3 | Av IC50 µg/mL | SD |
| 0 | 0 | 0 | 0 | 0 | 0 | 0 | 0 | 0.1135 | 0.0715 | 0.0945 | 0.093166667 | 0.021032 |
| 0.05 | 0.135 | 0.13 | 0.133 | 45.12195 | 47.15447 | 45.93496 | 1.023013 |  |  |  | IC50 µM | SD |
| 1 | 0.108 | 0.105 | 0.107 | 56.09756 | 57.31707 | 56.50407 | 0.620945 |  |  |  | 0.251516297 | 0.056778 |
| 5 | 0.095 | 0.0925 | 0.093 | 61.38211 | 62.39837 | 62.19512 | 0.537754 |  |  |  |  |  |
| 50 | 0.084 | 0.081 | 0.084 | 65.85366 | 67.07317 | 65.85366 | 0.704086 |  |  |  |  |  |
| 100 | 0.071 | 0.068 | 0.07 | 71.13821 | 72.35772 | 71.54472 | 0.620945 |  |  |  |  |  |
|  |  |  |  |  |  |  |  |  |  |  |  |  |
|  |  |  |  |  |  |  |  |  |  |  |  |  |
| LMH5 | Blank0.246 |  | MWt | 404.87 |  |  |  |  |  |  |  |  |
| Conc. µg/mL | Abc1 | Abc2 | Ab3 | ınh%1 | ınh%2 | ınh%3 | SD inh | IC50-1 | IC50-2 | IC50-3 | Av IC50 µg/mL | SD |
| 0 | 0 | 0 | 0 | 0 | 0 | 0 | 0 | 0.755 | 0.664 | 0.6 | 0.673 | 0.077891 |
| 0.05 | 0.155 | 0.145 | 0.143 | 36.99187 | 41.05691 | 41.86992 | 2.613455 |  |  |  | IC50 µM | SD |
| 1 | 0.117 | 0.113 | 0.1135 | 52.43902 | 54.06504 | 53.86179 | 0.885955 |  |  |  | 1.662261961 | 0.192385 |
| 5 | 0.105 | 0.101 | 0.099 | 57.31707 | 58.94309 | 59.7561 | 1.24189 |  |  |  |  |  |
| 50 | 0.101 | 0.095 | 0.092 | 58.94309 | 61.38211 | 62.60163 | 1.862836 |  |  |  |  |  |
| 100 | 0.095 | 0.084 | 0.0835 | 61.38211 | 65.85366 | 66.05691 | 2.642276 |  |  |  |  |  |
|  |  |  |  |  |  |  |  |  |  |  |  |  |
|  |  |  |  |  |  |  |  |  |  |  |  |  |
| LMH6 | Blank0.246 |  | MWt | 366.48 |  |  |  |  |  |  |  |  |
| Conc. µg/mL | Abc1 | Abc2 | Ab3 | ınh%1 | ınh%2 | ınh%3 | SD inh | IC50-1 | IC50-2 | IC50-3 | Av IC50 µg/mL | SD |
| 0 | 0 | 0 | 0 | 0 | 0 | 0 | 0 | 0.0745 | 0.048 | 0.082 | 0.068166667 | 0.017863 |
| 0.05 | 0.1235 | 0.122 | 0.125 | 49.79675 | 50.4065 | 49.18699 | 0.609756 |  |  |  | IC50 µM | SD |
| 1 | 0.103 | 0.095 | 0.108 | 58.13008 | 61.38211 | 56.09756 | 2.665625 |  |  |  | 0.186003784 | 0.048742 |
| 5 | 0.095 | 0.088 | 0.099 | 61.38211 | 64.22764 | 59.7561 | 2.263319 |  |  |  |  |  |
| 50 | 0.085 | 0.081 | 0.087 | 65.44715 | 67.07317 | 64.63415 | 1.24189 |  |  |  |  |  |
| 100 | 0.064 | 0.06 | 0.069 | 73.98374 | 75.60976 | 71.95122 | 1.833028 |  |  |  |  |  |
|  |  |  |  |  |  |  |  |  |  |  |  |  |
| LMH7 | Blank0.246 |  | MWt | 370.42 |  |  |  |  |  |  |  |  |
| Conc. µg/mL | Abc1 | Abc2 | Ab3 | ınh%1 | ınh%2 | ınh%3 | SD inh | IC50-1 | IC50-2 | IC50-3 | Av IC50 µg/mL | SD |
| 0 | 0 | 0 | 0 | 0 | 0 | 0 | 0 | 0.0745 | 0.1065 | 0.065 | 0.082 | 0.021743 |
| 0.05 | 0.124 | 0.128 | 0.1235 | 49.5935 | 47.96748 | 49.79675 | 1.002618 |  |  |  | IC50 µM | SD |
| 1 | 0.104 | 0.11 | 0.102 | 57.72358 | 55.28455 | 58.53659 | 1.692411 |  |  |  | 0.221370336 | 0.058698 |
| 5 | 0.097 | 0.105 | 0.095 | 60.56911 | 57.31707 | 61.38211 | 2.151017 |  |  |  |  |  |
| 50 | 0.087 | 0.098 | 0.082 | 64.63415 | 60.1626 | 66.66667 | 3.327379 |  |  |  |  |  |
| 100 | 0.069 | 0.078 | 0.067 | 71.95122 | 68.29268 | 72.76423 | 2.381896 |  |  |  |  |  |
|  |  |  |  |  |  |  |  |  |  |  |  |  |
| LMH9 | Blank0.246 |  | MWt | 370.42 |  |  |  |  |  |  |  |  |
| Conc. µg/mL | Abc1 | Abc2 | Ab3 | ınh%1 | ınh%2 | ınh%3 | SD inh | IC50-1 | IC50-2 | IC50-3 | Av IC50 µg/mL | SD |
| 0 | 0 | 0 | 0 | 0 | 0 | 0 | 0 | 0.2245 | 0.2015 | 0.261 | 0.229 | 0.030004 |
| 0.05 | 0.128 | 0.127 | 0.13 | 47.96748 | 48.37398 | 47.15447 | 0.620945 |  |  |  | IC50 µM | SD |
| 1 | 0.115 | 0.113 | 0.118 | 53.25203 | 54.06504 | 52.03252 | 1.023013 |  |  |  | 0.618217159 | 0.081 |
| 5 | 0.111 | 0.106 | 0.115 | 54.87805 | 56.91057 | 53.25203 | 1.833028 |  |  |  |  |  |
| 50 | 0.102 | 0.097 | 0.109 | 58.53659 | 60.56911 | 55.69106 | 2.45029 |  |  |  |  |  |
| 100 | 0.095 | 0.084 | 0.102 | 61.38211 | 65.85366 | 58.53659 | 3.688525 |  |  |  |  |  |
|  |  |  |  |  |  |  |  |  |  |  |  |  |
| Trolox | Blank0.246 |  | MWt | 250.29 |  |  |  |  |  |  |  |  |
| Conc. µg/mL | Abc1 | Abc2 | Ab3 | ınh%1 | ınh%2 | ınh%3 | SD inh | IC50-1 | IC50-2 | IC50-3 | Av IC50 µg/mL | SD |
| 0 | 0 | 0 | 0 | 0 | 0 | 0 | 0 | 1.02 | 0.74 | 0.565 | 0.775 | 0.22951 |
| 0.05 | 0.142 | 0.135 | 0.128 | 42.27642 | 45.12195 | 47.96748 | 2.845528 |  |  |  | IC50 µM | SD |
| 1 | 0.1235 | 0.119 | 0.115 | 49.79675 | 51.62602 | 53.25203 | 1.728638 |  |  |  | 3.096408167 | 0.916978 |
| 5 | 0.111 | 0.105 | 0.101 | 54.87805 | 57.31707 | 58.94309 | 2.046026 |  |  |  |  |  |
| 50 | 0.085 | 0.081 | 0.078 | 65.44715 | 67.07317 | 68.29268 | 1.427595 |  |  |  |  |  |
| 100 | 0.045 | 0.039 | 0.035 | 81.70732 | 84.14634 | 85.77236 | 2.046026 |  |  |  |  |  |
