# Supplementary material for: Thiazole-carboxamide derivatives as potent antioxidant agents with drug-like properties: In vitro, molecular docking, and DFT studies
Source: PLoS One. 2025 Sep 19;20(9):e0331000. doi: 10.1371/journal.pone.0331000 (PMC12448349; doi:10.1371/journal.pone.0331000)
Supplement: S2 Table — (DOCX) [file pone.0331000.s003.docx]

**S2 Table. The raw data for alpha amylase assay with used different concentrations for all evaluated compounds**

| Conc. | LMH1 | LMH2 | LMH3 | LMH4 | LMH5 | LMH6 | LMH7 | LMH10 | Acarbose |
| --- | --- | --- | --- | --- | --- | --- | --- | --- | --- |
| 0 | 0 | 0 | 0 | 0 | 0 | 0 | 0 | 0 | 0 |
| 5 | 31.91126 | 41.80887 | 20.98976 | 29.18089 | 34.30034 | 18.25939 | 19.96587 | 28.83959 | 53.22 |
| 50 | 40.10239 | 42.66212 | 23.37884 | 32.25256 | 35.83618 | 20.98976 | 23.20819 | 34.30034 | 54.91 |
| 100 | 41.80887 | 47.95222 | 27.13311 | 37.71331 | 36.34812 | 22.69625 | 24.74403 | 37.03072 | 66.1 |
